# The Short- and Long-Term Risk of Mortality in Intracranial Hemorrhage Patients with Tranexamic Acid Treatment in a Population-Based Cohort Study

**DOI:** 10.3390/jcm13061597

**Published:** 2024-03-11

**Authors:** Chien-Ming Chiu, Sung-Yuan Hu, Pei-Lun Liao, Jing-Yang Huang, Ming-Chih Chou, Shun-Fa Yang, Chao-Bin Yeh

**Affiliations:** 1 Institute of Medicine, Chung Shan Medical University, Taichung 402, Taiwan; 2 Department of Emergency Medicine, Taichung Veterans General Hospital, Taichung 407, Taiwan; 3 School of Medicine, Chung Shan Medical University, Taichung 402, Taiwan; 4 Department of Post-Baccalaureate Medicine, College of Medicine, National Chung Hsing University, Taichung 402, Taiwan; 5 School of Medicine, National Yang Ming Chiao Tung University, Taipei 112, Taiwan; 6 Department of Medical Research, Chung Shan Medical University Hospital, Taichung 402, Taiwan; 7 Department of Surgery, Chung Shan Medical University Hospital, Taichung 402, Taiwan; 8 Department of Emergency Medicine, School of Medicine, Chung Shan Medical University, Taichung 402, Taiwan; 9 Department of Emergency Medicine, Chung Shan Medical University Hospital, Taichung 402, Taiwan

**Keywords:** tranexamic acid, intracranial hemorrhage, mortality, thromboembolic events, cohort study

## Abstract

**Background:** The mortality rate associated with nontraumatic intracranial hemorrhage (NTICrH) remains consistently high under the current care modality. The effectiveness of tranexamic acid (TXA) as a treatment option is still a subject of debate. This study aims to assess the association between TXA administration and both short-term and long-term mortality rates in patients with NTICrH. **Methods:** We conducted a retrospective cohort study using data from the Taiwan National Health Insurance Research Database (NHIRD) spanning from January 2000 to December 2017. The study population consists of NTICrH patients admitted to the ICU, divided into two groups: patients who were treated with TXA and those who were not. Propensity score matching (PSM) was conducted to balance the baseline characteristics of the two groups. Cox proportional hazard analysis was conducted to estimate the hazard ratio (HR) for the all-cause mortality. Sensitivity analyses were performed using the inverse probability of treatment-weighted hazard ratio (IPTW-HR). To assess the timing of TXA use, we compared the risk of all-cause mortality within 180 days between patients receiving early TXA treatment and those receiving late TXA treatment. **Results:** There was no significant difference in 180-day all-cause mortality between the groups; the hazard ratio was 1.07 (95% CI: 0.96–1.20) in patients treated with TXA compared to those without TXA treatment. Within 7 days of admission, patients treated with TXA had a lower hazard ratio of 0.81 (95% CI: 0.74–0.90) for all-cause mortality. **Conclusions:** Lower mortality within the first 7 days was observed in patients with NTICrH who received TXA.

## 1. Introduction

Nontraumatic intracranial hemorrhage (NTICrH) is a major cause of disability and mortality worldwide. NTICrH mainly consists of spontaneous intracerebral hemorrhage (ICH) and spontaneous subarachnoid hemorrhage (SAH). NTICrH primarily causes brain tissue injury through hematoma formation, perifocal edema development, and increased intracranial pressure. Progression of hemorrhage, hematoma expansion, and rebleeding are associated with neurologic deterioration and adverse outcomes. Thus, the early prevention of hemorrhage is crucial [1].

Tranexamic acid (TXA) is an antifibrinolytic agent that inhibits the proteolytic activity of plasmin [2]. The early administration of TXA in patients with trauma [3] and women with postpartum hemorrhage [4] effectively reduces hemorrhage-related mortality. TXA also decreases blood loss and major bleeding outcomes in patients undergoing surgery [5,6]. However, the benefits of TXA in patients with NTICrH are uncertain.

Prompt hemostatic therapies potentially yield better outcomes in patients with nontraumatic intracranial hemorrhage, but evidence of improved outcomes for NTICrH after TXA administration is lacking. A systematic review and meta-analysis of 14 randomized controlled trials (RCTs) and one controlled clinical study, with a total of 4883 patients with NTICrH, demonstrated a significant decrease in mortality in patients with SAH (relative risk = 0.72, 95% confidence interval [CI]: 0.49 to 0.96, *p* < 0.001) [7]. By contrast, another meta-analysis of 7 RCTs, encompassing a total of 2917 patients with aneurysmal SAH, found that among those treated with TXA, there were no benefits in terms of the modified Rankin Scale score (Risk Difference [RD] = −0.01, 95% CI: −0.05 to 0.02, *p* = 0.51) or mortality (RD = 0.00, 95% CI: −0.03 to 0.04, *p* = 0.91) compared with the control group [8]. Two randomized placebo-controlled trials in patients with spontaneous ICH (TICH-2 trial [9] and STOP-AUST trial [10]) reported conflicting results regarding the preventive effect on hematoma expansion. Moreover, a recently published randomized placebo-controlled trial focused on Non-Vitamin K Antagonist Oral Anticoagulants (NOAC)-related ICH did not show better outcomes compared to those treated with TXA [11]. The roles of hemostatics (such as vitamin K, fresh frozen plasma) used in oral anticoagulant-related ICH remain poorly defined [12,13]. Furthermore, studies investigating both spontaneous ICH and SAH are uncommon. Therefore, this nationwide cohort study assessed the association between TXA administration and the mortality rate of patients with NTICrH in Taiwan.

## 2. Materials and Methods

### 2.1. Study Design and Source of Data

This retrospective cohort study utilized the Longitudinal Health Insurance Database 2000 (LHID2000) from January 2000 to December 2017 to investigate the association between TXA treatment and the risk of all-cause mortality in patients with intracranial hemorrhage admitted to intensive care units (ICUs). LHID2000 is a sub-dataset that comprised 2 million individuals randomly sampled from the National Health Insurance Research Database (NHIRD) [14], which contains insurance claim records from the National Health Insurance program since 1997. The National Health Insurance program covered over 99% of Taiwan’s population in the 2010s. According to the NHRI report, there were no significant differences in the distribution of gender, age, and healthcare costs between the LHID group and all insured enrollees [15]. Data files are anonymized and linked to encrypted identification numbers to protect patient privacy. Claims data include medical records of outpatient treatments and diagnoses, as well as documentation of hospital stays and emergency room records.

This study received approval from the Institutional Review Board of Chung Shan Medical University Hospital (approval number: CS2-20036). We submitted a research plan to the Health and Welfare Data Science Center of the Ministry of Health and Welfare and obtained authorization to access and analyze the data. All data underwent encryption and remained anonymous during the analysis, and patient consent was waived by the Institutional Review Board of Chung Shan Medical University Hospital.

### 2.2. Study Population

NTICrH was defined as a diagnosis using International Classification of Diseases, Ninth Revision, Clinical Modification (ICD-9-CM) codes 430, 431, and 432, as well as International Classification of Diseases, Tenth Revision, Clinical Modification (ICD-10-CM) codes I60, I61, and I62, from January 2001 to December 2016. We selected patients diagnosed with NTICrH who were admitted to the Intensive Care Unit (ICU). The ICU medical records were identified using specific National Health Insurance (NHI) codes: 02011, 02012, 02013, 03010, 03011, 03012, 03013, 03047, 03048, 03049, 03050, 05151, and 05152. The index date was defined as the date of admission for NTICrH. The exclusion criteria were as follows: (1) age less than 18 years on the index date (*n* = 123) and (2) death before the index date (*n* = 9). The remaining patients were divided into two groups based on their TXA treatment: the TXA group, comprising 7242 patients who received TXA, and the control group, comprising 10,857 patients who did not receive TXA. To assess the association between the timing of TXA use and all-cause mortality, we compared the patients who received early TXA treatment, defined as those who received TXA in the emergency departments (EDs), with the patients who received late TXA treatment, defined as those who received TXA after hospital admission.

To account for potential confounding effects from measured factors, we employed 1:1 propensity score matching (PSM) to balance the characteristics of sex, age (within ±1 year) on the index date, comorbidities, and medication usage between the TXA and control groups. PSM was conducted using the greedy nearest neighbor algorithm. Non-replacement matching with a caliper width of 0.1 was implemented using the PROC PSMATCH procedure in SAS software version 9.4 (SAS Institute, Cary, NC, USA).

### 2.3. Characteristics, Comorbidities, and Study Outcomes

We assessed the baseline demographic characteristics, comorbidities, medications, and received procedures within 180 days before the index date in both the TXA group and the control group. The comorbidities considered in the analysis included hypertension, diabetes mellitus, hyperlipidemia, kidney diseases, chronic pulmonary diseases, liver diseases, ischemic heart diseases, atrial fibrillation and flutter, congestive heart failure, malignancies, rheumatic diseases, dementia, peripheral vascular diseases, and peptic ulcer disease. Medications included non-steroidal anti-inflammatory drugs, proton-pump inhibitors (PPIs), histamine type-2 receptor antagonists (H2 blockers), aspirin, clopidogrel, ticagrelor, vitamin K, and other hemostatics, laxatives, furosemide, metoclopramide, magnesium oxide, antiepileptics, antibiotics, alpha-blockers, beta-blockers, calcium channel blockers, angiotensin-converting enzyme inhibitors (ACEIs), and angiotensin receptor blockers (ARBs). Procedures such as nasogastric tube feeding, ventilator usage, intracranial pressure monitoring, and cranial decompression surgery were also identified.

### 2.4. Outcome Measurement

The primary outcome of this study was all-cause mortality within 180 days following the index date. Secondary outcomes included the diagnosis of thromboembolic events, specifically venous events such as deep vein thrombosis and pulmonary embolism, as well as arterial events including acute myocardial infarction and ischemic stroke, within 180 days following the index date. All patients included in this study were followed from the index date until their withdrawal from the National Health Insurance program, the diagnosis of a study event, or 180 days after the index date.

### 2.5. Statistical Analysis

Descriptive statistics were utilized to examine the distribution of baseline characteristics. The absolute standardized difference (ASD) was used to assess the covariate differences between the two groups. An ASD value of <0.10 indicates balanced characteristics between the study groups. The incidence density and 95% confidence interval (CI) of study events were calculated using the normal approximation to the Poisson distribution [16]. The relative risk and 95% CI were calculated using Poisson regression. The cumulative probability of mortality was assessed using Kaplan–Meier (K–M) analysis, and the difference in K–M curves between the study groups was evaluated using a log-rank test. After testing the proportional hazards assumption, Cox proportional hazard analysis was conducted to estimate the hazard ratio (HR) for mortality. The follow-up time was divided into three periods (1–7, 8–14, and 15–180 days) to investigate the immediate, short-term, and long-term associations between TXA administration and the risk of mortality or thromboembolic events.

We performed a sensitivity analysis using the inverse probability of treatment-weighted hazard ratio (IPTW-HR). This approach enabled us to address the imbalance between the treatment groups and minimize the influence of potential confounding variables. By employing IPTW, our goal was to obtain more robust and reliable results in our analysis. All statistical analyses were conducted using SAS software version 9.4, and a significance level of *p* < 0.05 was considered statistically significant.

## 3. Results

### 3.1. Participant Characteristics

**Figure 1:**
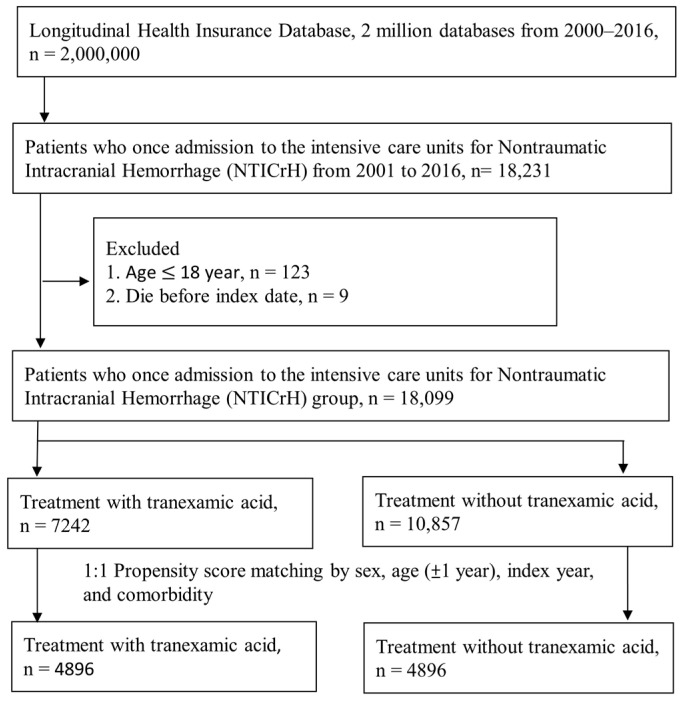
Study flowchart of selection participants.

We identified a total of 18,099 patients who had ICU admission with NTICrH. Among them, 7242 patients received TXA treatment, while 10,857 patients did not (Figure 1). In the TXA group, 62.47% of the patients were male, and more than 56% were aged ≥61 years. Before PSM, there were unbalanced baseline characteristics with an ASD > 0.10 between the two groups, including the index year, ventilator usage, and medications (such as PPIs, H2 blockers, vitamin K and other hemostatics, furosemide, antiepileptics, antibiotics, ACEIs, and ARBs). After PSM, each group consisted of 4896 patients, and the two groups were balanced in terms of each baseline characteristic, as indicated by an ASD of <0.10 for each covariate (Table 1).

### 3.2. Risk of 180-Day Mortality in Patients Treated with TXA

**Figure 2:**
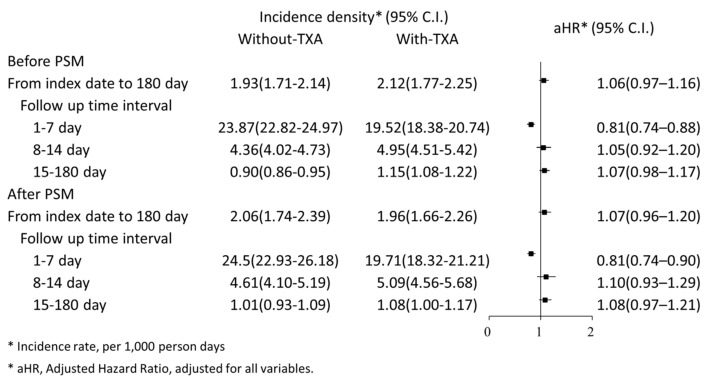
Incidence density and adjusted hazard ratio of mortality in NTICrH patients with TXA compared with NTICrH patients without TXA treatment stratified by follow up period.

**Figure 3:**
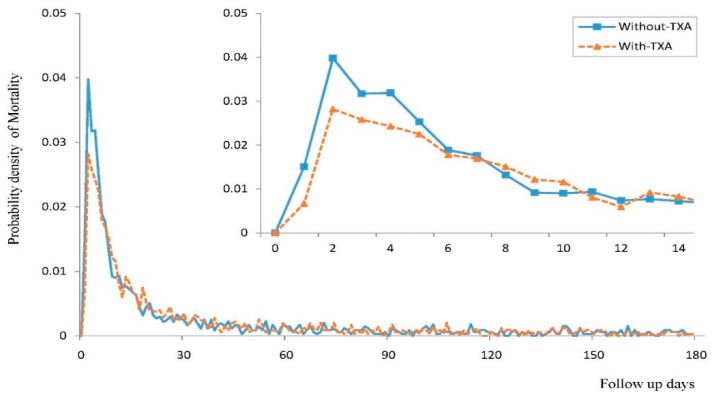
The daily probability density of mortality in NTICrH patients with TXA and NTICrH patients without TXA treatment.

**Figure 4:**
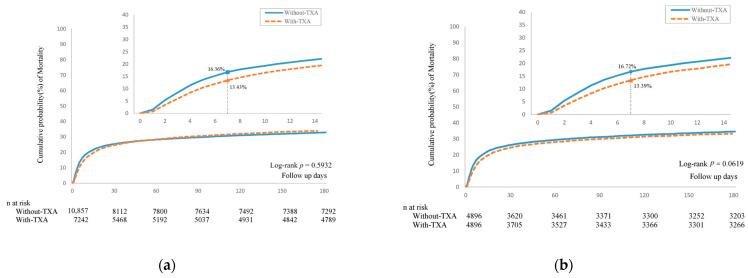
Kaplan–Meier curves for the 180 days mortality risk in NTICrH patients with TXA and NTICrH patients without TXA treatment. (**a**) Before PSM, (**b**) After PSM.

After PSM, the hazard ratio for the 180-day all-cause mortality is 1.07 (95% CI: 0.96–1.20) in patients treated with TXA compared with patients without TXA treatment. Within the first 7 days from the index date, the incidence density of mortality (per 1000 person-days) was 19.71 (95% CI: 18.32–21.21) in the TXA group and 24.50 (95% CI: 22.93–26.18) in the control group (Figure 2). As shown in Figure 3, compared to the control group, the TXA group exhibited a significantly lower mortality rate within 7 days, but the mortality rates from 7 to 180 days were similar between the two groups. K–M survival analysis did not demonstrate a significantly lower cumulative incidence of mortality within 180 days in the TXA group compared to the control group (log-rank test: *p* = 0.0619; Figure 4).

**Figure 5:**
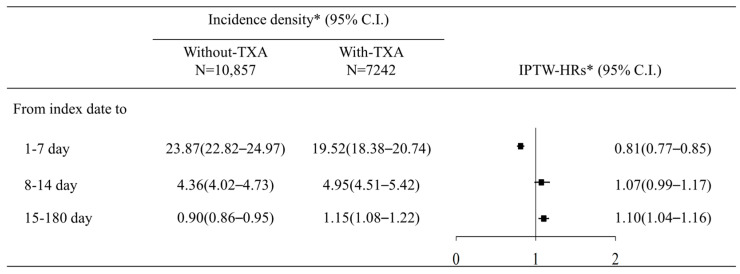
Sensitivity analysis of incidence density and inverse probability of treatment weighted hazard ratio of mortality in NTICrH patients with TXA compared with NTICrH patients without TXA treatment. * IPTW: inverse probability of treatment-weighted. * Incidence density: incidence rate, per 1000 person days., adjusted variable including age, sex, nasogastric tube feeding, ventilator usage, intracranial pressure monitor, intracranial decompression operation, comorbidities, and medication.

Multivariable Cox regression analysis was performed to estimate the adjusted hazard ratio (aHR). The TXA group exhibited a significantly reduced risk of mortality (aHR: 0.81, 95% CI: 0.74–0.90) compared to the control group during the period of 1 to 7 days. In the periods of 8 to 14 days and 15 to 180 days, the aHR for the TXA group was 1.10 (95% CI: 0.93–1.29) and 1.08 (95% CI: 0.97–1.21), respectively (Figure 2). The result of IPTW-HRs, presented in Figure 5, was consistent with the findings from the multivariable Cox regression analysis. Table 2 presents the factors associated with the risk of mortality.

### 3.3. Risk of 180-Day Mortality Compared between Early and Late TXA Treatment

We compared the risk of mortality in the patients with early and late TXA treatment with their matched control individuals (Table 3). Between 1 and 7 days, the early TXA treatment group had a lower adjusted hazard ratio (aHR) of 0.68 (95% CI: 0.55–0.84), while the late TXA treatment group had an aHR of 0.82 (95% CI: 0.74–0.90). There was no significant difference in all-cause mortality within 180 days between the groups of patients receiving early and late therapy. In the early-treated cohort, the hazard ratio for the 180-day all-cause mortality is 1.01 (95% CI: 0.78–1.33) in patients treated with TXA compared with patients without TXA treatment. In the late-treated cohort, the hazard ratio for the 180-day all-cause mortality is 1.07 (95% CI: 0.97–1.18) in patients treated with TXA compared with patients without TXA treatment.

## 4. Discussion

The results of this study revealed that TXA administration was significantly associated with decreased mortality within 7 days among patients with NTICrH who had been hospitalized in ICUs. No significant difference in mortality was noted from 7 to 180 days after the index date between the two groups. The occurrence of thromboembolic events was comparable between the two groups.

The mortality rate of spontaneous ICH has remained consistently high [17,18]. Nearly one-quarter of patients with spontaneous ICH have died within the first 7 days [19,20]. Although the fatality rate of spontaneous SAH has been reduced through early interventions such as surgical clipping or endovascular coiling [21], two-day fatality remains as high as 25% [22]. Greater research attention should be paid to short-term mortality. In the TICH-2 trial [9], although not statistically significant, fewer patients with spontaneous ICH in the TXA group died in the first week. In our study, the TXA group had a significantly lower risk of mortality within 7 days. High short-term mortality is potentially caused by early deterioration of bleeding. Hematoma expansion in ICH occurs in the early stage and is considered a major predictor of poor neurologic outcome and death [1,23]. Aneurysm rebleeding in SAH is also associated with high mortality [24], with an incidence rate of 10–22% within 24 h [8,25]. Therefore, the hemostatic effects of TXA that stop bleeding may reduce early mortality.

Numerous studies have investigated the relationship between TXA administration and mortality or bleeding control in patients with NTICrH. In the TICH-2 trial [9], the volume of hematoma expansion was significantly lower in the TXA group. In a large meta-analysis of RCTs involving spontaneous ICH patients, TXA was associated with a decreased risk of hematoma expansion but did not influence the three-month mortality [26]. A meta-analysis of ten RCTs investigating aneurysmal SAH demonstrated significantly lower rebleeding rates in patients receiving TXA [27]. However, no significant difference was observed in overall mortality between the two groups. Liu et al. conducted a meta-analysis of seven RCTs including patients with spontaneous SAH and reported that TXA reduced rebleeding rates but had no effect on mortality [8]. These large randomized trials and meta-analyses have reported that TXA administration is associated with improved bleeding control but had nonsignificant effects on mortality. These inconsistent findings regarding mortality may be caused by differences in outcome assessment times; these studies were all conducted one month after the event rather than in the early stage.

To assess the association between earlier TXA use and all-cause mortality, we compared the HR of mortality of patients at EDs who had been administered TXA with the HR of those who had not received TXA. Significantly lower aHRs were observed in the early treatment cohort compared with the late treatment cohort. Among all patients, the lowest mortality was observed in patients who received TXA both in EDs and after hospitalization. In addition to its benefits, the safety of TXA has been explored in numerous studies. In a meta-analysis, Hu et al. observed a similar rate of thromboembolism between the TXA and control groups comprising patients with traumatic and spontaneous intracranial hemorrhage [24]. In the TICH-2 trial [9], no increase in the occurrence of thromboembolic events was observed in patients with ICH who received TXA. For spontaneous SAH, the ULTRA trial [28] reported similar rates of thromboembolic complications during endovascular treatment regardless of TXA use. These results are consistent with the present study findings on side effects.

The database LHID used in this study was a larger sample randomly drawn from NHIRD, so the results are likely to be accurate and reliable. However, some limitations of this study should be acknowledged. First, the severity of the disease is not reported in the NHIRD. No record of the initial consciousness level was available. For patients with intracranial hemorrhage, initial consciousness is a crucial predictor of disease severity. To account for this limitation, this study only included patients who had been admitted to the ICU, and characteristics such as ventilator use, intracranial pressure monitoring, and intracranial decompression operation were matched to obtain a sample with relatively homogeneous severity. Second, the NHIRD does not contain data on the duration of TXA administration. Based on the timing of TXA administration, we stratified the study population into early (in the emergency department) and late (after admission) treatment cohorts to analyze the association between early TXA treatment and the risk of mortality. Significantly lower mortality rates were observed in the early TXA group compared to the late TXA group. Third, we proposed to differentiate between various types of intracranial hemorrhages such as ICH, subarachnoid hemorrhage, subdural hematoma, and epidural hematoma. However, further categorization leads to a reduction in sample size, thereby diminishing the statistical power of the tests. Additionally, extensive research has already been conducted on different categories of intracranial hemorrhage. The diagnosis of non-aneurysmal intracranial hemorrhage is less common in NTICrH cases, and clinically, the diagnosis often only states subarachnoid hemorrhage without distinguishing between aneurysmal or non-aneurysmal origins. Therefore, this study was unable to effectively differentiate between aneurysmal and non-aneurysmal cases for comparison. However, numerous large RCTs and meta-analyzes have demonstrated that TXA administration can decrease expansion in patients with spontaneous ICH, as well as rebleeding in patients with spontaneous SAH [8,9,26,29]. Therefore, mortality was selected as the primary outcome of interest rather than the bleeding control demonstrated by imaging. Fourthly, it is crucial to consider that the efficacy and safety of tranexamic acid in patients with intracerebral hemorrhage (ICH) could potentially be influenced by interactions with other treatments, notably anticoagulant therapy [11]. However, our study did not include NOAC treatment as a variable, thereby limiting our ability to assess this interaction effect. Future studies should aim to incorporate the evaluation of tranexamic acid in conjunction with anticoagulant treatments to fully understand the potential interaction effects. Finally, the sample size was considerably reduced following PSM for matching. However, the results were deemed unbiased because the groups had a similar distribution of baseline characteristics before and after PSM (Table 1). We also performed a IPTW analysis (Figure 5), and the finding is similar to the results of PSM.

## 5. Conclusions

Lower mortality within the first 7 days was observed in patients with NTICrH who received TXA. Given the various limitations inherent in observational studies, we suggest treating this study as a preliminary step for future prospective research. Further studies are necessary to develop more comprehensive and prospective investigations, with the goal of validating and expanding upon our initial findings.

## Figures and Tables

**Table 1:** Baseline characteristics among study groups.

Variables	Before PSM			After PSM		
	Without-TXA	With-TXA	ASD	Without-TXA	With-TXA	ASD
N	10,857	7242		4896	4896	
Index year			0.213			0.024
2001–2005	3431 (31.6%)	1794 (24.77%)		1291 (26.37%)	1266 (25.86%)	
2006–2010	3529 (32.5%)	2206 (30.46%)		1470 (30.02%)	1505 (30.74%)	
2011–2016	3897 (35.89%)	3242 (44.77%)		2135 (43.61%)	2125 (43.4%)	
Sex			0.055			0.007
Female	4366 (40.21%)	2718 (37.53%)		1869 (38.17%)	1886 (38.52%)	
Male	6491 (59.79%)	4524 (62.47%)		3027 (61.83%)	3010 (61.48%)	
Age			0.062			0.043
19–50	2357 (21.71%)	1500 (20.71%)		1007 (20.57%)	999 (20.4%)	
51–60	2370 (21.83%)	1612 (22.26%)		1120 (22.88%)	1089 (22.24%)	
61–70	2239 (20.62%)	1486 (20.52%)		992 (20.26%)	1006 (20.55%)	
71–80	2395 (22.06%)	1542 (21.29%)		1043 (21.3%)	1080 (22.06%)	
>80	1496 (13.78%)	1102 (15.22%)		734 (14.99%)	722 (14.75%)	
Charlson Comorbidity Index			0.062			0.042
1	4470 (41.17%)	2839 (39.2%)		1976 (40.36%)	1931 (39.44%)	
2	2377 (21.89%)	1552 (21.43%)		1067 (21.79%)	1039 (21.22%)	
3	4010 (36.93%)	2851 (39.37%)		1853 (37.85%)	1926 (39.34%)	
Length of hospital stay			0.094			0.089
1–7 day	3294 (30.34%)	1884 (26.01%)		1479 (30.21%)	1295 (26.45%)	
8–14 day	2219 (20.44%)	1417 (19.57%)		933 (19.06%)	976 (19.93%)	
>=15 day	5344 (49.22%)	3941 (54.42%)		2484 (50.74%)	2625 (53.62%)	
Nasogastric tube feeding			0.094			0.011
No	3379 (31.12%)	1945 (26.86%)		1396 (28.51%)	1371 (28%)	
Yes	7478 (68.88%)	5297 (73.14%)		3500 (71.49%)	3525 (72%)	
Ventilator usage			0.142			0.014
No	1135 (10.45%)	471 (6.5%)		392 (8.01%)	374 (7.64%)	
Yes	9722 (89.55%)	6771 (93.5%)		4504 (91.99%)	4522 (92.36%)	
Intracranial pressure monitor			0.050			0.006
No	7719 (71.1%)	4984 (68.82%)		3434 (70.14%)	3421 (69.87%)	
Yes	3138 (28.9%)	2258 (31.18%)		1462 (29.86%)	1475 (30.13%)	
Intracranial decompression operation			0.065			0.005
No	7985 (73.55%)	5115 (70.63%)		3558 (72.67%)	3546 (72.43%)	
Yes	2872 (26.45%)	2127 (29.37%)		1338 (27.33%)	1350 (27.57%)	
Co-morbidity						
Hypertension	7961 (73.33%)	5389 (74.41%)	0.025	3635 (74.24%)	3643 (74.41%)	0.004
Diabetes mellitus	2826 (26.03%)	1862 (25.71%)	0.007	1289 (26.33%)	1284 (26.23%)	0.002
Hyperlipidemia	1478 (13.61%)	938 (12.95%)	0.019	648 (13.24%)	661 (13.5%)	0.008
Kidney diseases	1146 (10.56%)	801 (11.06%)	0.016	553 (11.29%)	542 (11.07%)	0.007
Chronic pulmonary diseases	1252 (11.53%)	847 (11.7%)	0.005	563 (11.5%)	588 (12.01%)	0.016
Liver diseases	1180 (10.87%)	880 (12.15%)	0.040	573 (11.7%)	575 (11.74%)	0.001
Ischemic heart diseases	1340 (12.34%)	830 (11.46%)	0.027	533 (10.89%)	567 (11.58%)	0.022
Atrial fibrillation and flutter	577 (5.31%)	300 (4.14%)	0.055	222 (4.53%)	227 (4.64%)	0.005
Congestive heart failure	948 (8.73%)	592 (8.17%)	0.020	413 (8.44%)	414 (8.46%)	0.001
Malignancies	721 (6.64%)	513 (7.08%)	0.018	355 (7.25%)	326 (6.66%)	0.023
Rheumatic diseases	104 (0.96%)	72 (0.99%)	0.004	46 (0.94%)	48 (0.98%)	0.004
Dementia	530 (4.88%)	385 (5.32%)	0.020	250 (5.11%)	245 (5%)	0.005
Peripheral vascular diseases	543 (5%)	296 (4.09%)	0.044	224 (4.58%)	223 (4.55%)	0.001
Peptic ulcer disease	1276 (11.75%)	939 (12.97%)	0.037	614 (12.54%)	614 (12.54%)	0.001
Medication						
NSAIDs	8129 (74.87%)	5430 (74.98%)	0.002	3663 (74.82%)	3664 (74.84%)	0.001
PPI	4008 (36.92%)	3122 (43.11%)	0.127	2012 (41.09%)	1970 (40.24%)	0.017
H2 blockers	6527 (60.12%)	5094 (70.34%)	0.216	3302 (67.44%)	3343 (68.28%)	0.018
Aspirin	3906 (35.98%)	2468 (34.08%)	0.040	1698 (34.68%)	1728 (35.29%)	0.013
Clopidogrel/Ticagrelor	621 (5.72%)	312 (4.31%)	0.065	226 (4.62%)	235 (4.8%)	0.009
Vitamin K and other hemostatics	1810 (16.67%)	4107 (56.71%)	0.913	1789 (36.54%)	1777 (36.29%)	0.005
Laxatives	8828 (81.31%)	6025 (83.2%)	0.049	4025 (82.21%)	4037 (82.46%)	0.006
Furosemide	3623 (33.37%)	2834 (39.13%)	0.120	1810 (36.97%)	1812 (37.01%)	0.001
Metoclopramide	4489 (41.35%)	3137 (43.32%)	0.040	2071 (42.3%)	2074 (42.36%)	0.001
Magnesium oxide	3111 (28.65%)	2110 (29.14%)	0.011	1344 (27.45%)	1352 (27.61%)	0.004
Antiepileptics	5473 (50.41%)	4170 (57.58%)	0.144	2708 (55.31%)	2696 (55.07%)	0.005
Antibiotics	1728 (15.92%)	877 (12.11%)	0.110	635 (12.97%)	623 (12.72%)	0.007
Alpha-blockers	1160 (10.68%)	869 (12%)	0.041	570 (11.64%)	565 (11.54%)	0.003
Beta-blockers	6999 (64.47%)	4769 (65.85%)	0.029	3199 (65.34%)	3183 (65.01%)	0.007
CCBs	8736 (80.46%)	6069 (83.8%)	0.087	4071 (83.15%)	4056 (82.84%)	0.008
ACEIs	3230 (29.75%)	1780 (24.58%)	0.116	1271 (25.96%)	1249 (25.51%)	0.010
ARBs	3868 (35.63%)	2988 (41.26%)	0.116	1955 (39.93%)	1965 (40.13%)	0.004

ACEIs: angiotensin converting enzyme inhibitors; ARBs: angiotensin receptor blockers; ASD: absolute standardized difference; CCBs: calcium channel blockers; H2-blockers: histamine type-2 receptor antagonists; NSAIDs: non-steroidal anti-inflammatory drugs; PPI: proton-pump inhibitors; PSM: propensity score matching; TXA: tranexamic acid.

**Table 2:** Multiple Cox regression to estimate the hazard ratio for the 180 days mortality risk.

Variable	aHR (95% CI)	
	1–7 Day	8–180 Day
Study group		
Without-TXA	Reference	Reference
With-TXA	0.81 (0.74–0.90)	1.09 (0.99–1.19)
Index year		
2001–2005	Reference	Reference
2006–2010	0.93 (0.80–1.08)	0.96 (0.82–1.13)
2011–2016	0.98 (0.84–1.14)	1.05 (0.90–1.23)
Sex		
Female	Reference	Reference
Male	1.15 (1.04–1.28)	0.95 (0.86–1.04)
Age		
19–50	Reference	Reference
51–60	0.79 (0.68–0.93)	1.09 (0.93–1.29)
61–70	0.85 (0.72–1.00)	1.22 (1.03–1.44)
71–80	1.07 (0.91–1.26)	1.37 (1.17–1.61)
>80	1.08 (0.91–1.29)	1.79 (1.51–2.12)
Nasogastric tube feeding (ref: no)	2.65 (2.31–3.05)	5.97 (4.84–7.36)
Ventilator usage (ref: no)	2.50 (1.89–3.33)	2.45 (1.70–3.52)
Intracranial pressure monitor (ref: no)	0.73 (0.64–0.83)	1.08 (0.97–1.19)
Intracranial decompression operation (ref: no)	0.48 (0.41–0.56)	0.70 (0.63–0.78)
Co-morbidity (ref: non)		
Hypertension	1.33 (1.17–1.51)	0.89 (0.79–1.00)
Diabetes mellitus	1.08 (0.96–1.21)	1.29 (1.17–1.44)
Hyperlipidemia	0.96 (0.81–1.13)	0.84 (0.72–0.97)
Kidney diseases	1.64 (1.42–1.89)	1.90 (1.68–2.15)
Chronic pulmonary diseases	0.94 (0.79–1.10)	1.04 (0.91–1.18)
Liver diseases	1.23 (1.06–1.42)	1.29 (1.13–1.47)
Ischemic heart diseases	1.40 (1.20–1.64)	1.07 (0.92–1.23)
Atrial fibrillation and flutter	1.00 (0.79–1.27)	0.78 (0.63–0.95)
Congestive heart failure	1.32 (1.11–1.58)	1.24 (1.07–1.44)
Malignancies	1.42 (1.19–1.70)	1.69 (1.47–1.94)
Rheumatic diseases	1.38 (0.89–2.13)	1.51 (1.01–2.25)
Dementia	0.95 (0.76–1.20)	0.91 (0.75–1.09)
Peripheral vascular diseases	0.93 (0.72–1.21)	0.90 (0.73–1.12)
Peptic ulcer disease	1.14 (0.98–1.33)	1.06 (0.94–1.21)
Medication (ref: non)		
NSAIDs	0.83 (0.75–0.92)	1.07 (0.95–1.20)
PPI	0.96 (0.85–1.08)	1.25 (1.13–1.39)
H2 blockers	1.00 (0.90–1.12)	1.04 (0.94–1.15)
Aspirin	1.12 (1.00–1.25)	1.06 (0.96–1.17)
Clopidogrel/Ticagrelor	0.98 (0.76–1.25)	0.97 (0.80–1.18)
Vitamin K and other hemostatic	1.16 (1.05–1.29)	1.15 (1.04–1.27)
Laxatives	0.21 (0.19–0.24)	0.58 (0.49–0.68)
Furosemide	0.72 (0.63–0.81)	1.65 (1.49–1.83)
Metoclopramide	0.76 (0.67–0.85)	1.30 (1.17–1.43)
Magnesium oxide	0.57 (0.50–0.66)	0.80 (0.72–0.89)
Antiepileptics	0.92 (0.83–1.02)	1.00 (0.90–1.10)
Antibiotics	0.85 (0.70–1.02)	1.10 (0.93–1.31)
Alpha-blockers	0.80 (0.65–0.99)	0.84 (0.73–0.98)
Beta-blockers	1.02 (0.91–1.14)	0.86 (0.78–0.96)
CCBs	0.68 (0.60–0.76)	0.77 (0.67–0.88)
ACEIs	0.62 (0.54–0.71)	0.91 (0.81–1.01)
ARBs	0.55 (0.48–0.62)	0.69 (0.62–0.77)

ACEIs: angiotensin converting enzyme inhibitors; aHR: adjusted hazard ratio; ARBs: angiotensin receptor blockers; CCBs: calcium channel blockers; H2-blockers: histamine type-2 receptor antagonists; NSAIDs: non-steroidal anti-inflammatory drugs; PPI: proton-pump inhibitors; TXA: tranexamic acid.

**Table 3:** Incidence density and hazard ratios of mortality in TXA early treated NTICrH patients compared with TXA late treated NTICrH patients.

	Incidence Density (95% CI)		HR * (95% CI)	aHR (95% CI)
	Without-TXA	With-TXA		
Early treatment				
From index date to 180 day	1.82 (1.51–2.20)	1.64 (1.42–1.90)	1.06 (0.82–1.37)	1.01 (0.78–1.33)
Follow up time interval				
1–7 day	25.08 (21.51–29.24)	18.41 (16.16–20.96)	0.74 (0.6–0.9)	0.68 (0.55–0.84)
8–14 day	3.48 (2.53–4.78)	4.2 (3.41–5.17)	1.21 (0.83–1.77)	1.14 (0.77–1.7)
15–180 day	0.77 (0.63–0.95)	0.82 (0.71–0.95)	1.07 (0.83–1.37)	1.02 (0.78–1.33)
Late treatment				
From index date to 180 day	1.95 (1.83–2.05)	2.13 (1.98–2.29)	1.32 (1.21–1.45)	1.07 (0.97–1.18)
Follow up time interval				
1–7 day	23.76 (22.67–24.91)	19.85 (18.54–21.25)	0.84 (0.77–0.91)	0.82 (0.74–0.9)
8–14 day	4.44 (4.08–4.82)	5.17 (4.67–5.73)	1.17 (1.02–1.33)	1.04 (0.9–1.21)
15–180 day	0.92 (0.86–0.97)	1.25 (1.17–1.34)	1.35 (1.24–1.48)	1.08 (0.98–1.19)

* Incidence density: incidence rate, per 1000 person days. Early treatment: with tranexamic acid use in emergency departments. Late treatment: without tranexamic acid use in emergency departments. aHR: adjusted hazard ratio, adjusted variable including age, sex, nasogastric tube feeding, ventilator usage, intracranial pressure monitor, intracranial decompression operation, comorbidities, and medication.

## Data Availability

Restrictions apply to the availability of these data. Data were obtained from the National Health Insurance database and are available from the authors with the permission of the National Health Insurance Administration of Taiwan.
